# 

**Published:** 1890-03

**Authors:** 


### TABLE OF CONTENTS.

ORIGINAL COMMUNICATIONS. PAGE

The Relations of Dentistry to Medicine, and Aseptic Dentistry. By J. S. Wight, M.D. . . . . . 129

Rapid Method of Inserting and Finishing Contour Fillings without the Aid of a Matrix. By J. E. Waitt, D.M.D. . . . . . 136

Oral Surgery Clinic. By Prof. John S. Marshall, M.D. . . . . . 139

REPORTS OF SOCIETY MEETINGS.

American Academy of Dental Science . . . . . . 141

Union Meeting of the Connecticut Valley Dental Society, the New England Dental Society, and the Connecticut State Dental Association, at Springfield, Mass., October 23, 24, and 25, 1889 . . . . . . 148

Seventh Annual Session of the Maryland State Dental Association . . . 168

EDITORIAL.

The National Association of Dental Faculties . . . . . . 182

American Dental Association . . . . . . 184

A Call for an International Dental Congress . . . . . . 185

FOREIGN CORRESPONDENCE . . . . . . 186

DOMESTIC CORRESPONDENCE . . . . . . 188

CURRENT NEWS.

Chicago and Vicinity . . . . . . 191

Items . . . . . . 192

